# Formulation and Evaluation of Nitrendipine Buccal Films

**DOI:** 10.4103/0250-474X.45402

**Published:** 2008

**Authors:** M. Nappinnai, R. Chandanbala, R. Balaijirajan

**Affiliations:** Department of Pharmaceutics, C. L. Baid Mehta College of Pharmacy, Jyothi Nagar, Thoraipakkam, Old Mahabalipuram Road, Chennai-600 096, India

**Keywords:** Buccal films, carboxymethylcellulose, hydroxylpropylcellulose, nitrendipine

## Abstract

A mucoadhesive drug delivery system for systemic delivery of nitrendipine, a calcium channel blocker through buccal route was formulated. Mucoadhesive polymers like hydroxypropylmethylcellulose K-100, hydroxypropylcellulose, sodium carboxymethylcellulose, sodium alginate, polyvinyl alcohol, polyvinyl pyrrolidone K-30 and carbopol-934P were used for film fabrication. The films were evaluated for their weight, thickness, percentage moisture absorbed and lost, surface pH, folding endurance, drug content uniformity, *In vitro* residence time, *In vitro* release and *ex vivo* permeation. Based on the evaluation of these results, it was concluded that buccal films made of hydroxylpropylcellulose and sodium carboxymethylcellulose (5±2% w/v; F-4), which showed moderate drug release (50% w/w at the end of 2 h) and satisfactory film characteristics could be selected as the best among the formulations studied.

Mucoadhesive drug delivery systems may be formulated to adhere to the mucosa of eyes, nose, oral (buccal), intestine, rectum and vagina^1^. Among these systems, the buccal mucosa offers many advantages like relatively large surface area of absorption, easy accessibility, simple delivery devices, avoiding hepatic first pass metabolism and feasibility of controlled drug delivery^2^. Buccal films are flexible, comfortable compared to the tablets and can circumvent shorter residence time of oral gels^3^. Few drugs that have been attempted as buccal films were nifedipine, isosorbide dinitrate, diltiazem hydrochloride and propranolol hydrochloride^4^. Nitrendipine is a calcium channel blocker used in the treatment of mild to moderate hypertension, chronic stable angina pectoris and Prinz metal's variant angina^5^. It undergoes extensive hepatic first pass metabolism and its oral bioavailability is 11±5%^6^. Its duration of action ranges from 4-48 h. There is a need for an alternative route of administration. It is a potent molecule (20 mg once daily) with low molecular weight and lipid solubility. These drug characteristics favour its absorption via buccal route. Buccal delivery of nitrendipine may circumvent hepatic first pass metabolism to improve its bioavailability. Hence in this work fabrication of mucoadhesive buccal films of nitrendipine using bioadhesive polymers was executed.

Nitrendipine (NTD) was obtained as a gift sample from M/s. Camlin Ltd., Mumbai. Hydroxypropylmethylcellulose K-100 (HPMC K-100) and hydroxypropylcellulose (HPC) were purchased from Lab Chemicals, Chennai. Sodium carboxymethylcellulose (SCMC), Polyvinyl alcohol (PVA), Carbopol-934P (CP-934P) and sodium alginate were purchased from S. D. Fine Chem. Ltd., Mumbai. Polyvinylpyrrolidone K-30 (PVP K-30) was purchased from Central Drug House, New Delhi. All additives were used as such. Gift sample of nitrendipine was subjected to tests specified in monograph of British Pharmacopoeia 2004^7^. UV spectrophotometer used was Shimadzu 1601.

Placebo films were prepared and those exhibiting appreciable organoleptic properties like continuity, physical appearance and non-stickiness were selected for incorporating NTD. The composition of NTD buccal films was shown in Table 1. For F-1, F-2, F-3, F-4, F-6, F-7, calculated amounts of polymers were dissolved in half the volume of distilled water with continuous stirring using mechanical stirrer. For F-5, carbopol-934P was allowed to swell in one fourth volume of water^8^–^10^. To this, HPC was added and dissolved in sufficient quantity of water with stirring. In case of F-8, PVA was dissolved in half the quantity of hot water (temperature between 80-100°) with stirring^11^,^12^. In the preparation of F-9 and F-10, PVP K-30 was added to the cooled PVA solution^3^,^11^. Plasticizers glycerin, propylene glycol, PEG 400 and PEG 4000 (after melting) were added with continuous stirring and the final volume was adjusted with water. NTD was incorporated in quantity such that one cm^2^ film contained 10 mg. Final dispersion was stirred with mechanical stirrer. The bubble free medicated solutions were casted in clean dry glass moulds and allowed to dry in hot air oven at 50°, till dry flexible films were formed. HPC based films were air dried. Dried films cut and trimmed to one cm^2^ were packed in aluminum foil.

**TABLE 1:** COMPOSITION OF BUCCAL FILMS

Ingredients	F-1	F-2	F-3	F-4	F-5	F-6	F-7	F-8	F-9	F-10
HPMC K 100	2	3	-	-	-	-	-	-	-	-
HPC	-	-	10	5	5	-	-	-	-	-
Sodium alginate	-	-	-	-	-	5	2	-	-	-
PVA	-	-	-	-	-	-	-	3	3	2
SCMC	-	-	-	2	-	-	2	-	-	-
CP-934 P	-	-	-	-	1	-	-	-	-	-
PVP K-30	-	-	-	-	-	-	-	-	1	2
Glycerin	2.5	2.5	1	2.5	-	5	2.5	2.5	-	-

HPMC is hydroxypropylmethylcellulose, HPC is hydroxypropylcellulose, PVA is polyvinylalcohol SCMA is sodiumcarboxymethylcellulose, CP is carbopol and PVP is polyvinylpyrolidine

Appearance of the films was evaluated by observing the colour, elegance, stickiness and texture. Weight per square cm of films was measured using electronic balance (Remi) for five samples of each film and mean weight was calculated^3^. The thickness of each film was measured using electronic Vernier Calipers (Mitutoyo) at six different points^3^. Five such films were taken under each formulation for the test and mean was calculated. Percentage moisture absorbed and lost was evaluated by weighing films after they were placed in humidity chamber at 79.5±5% relative humidity for three days and reweighed. Percentage weight gain was calculated as moisture absorbed^13^. Films placed in dessicator containing anhydrous calcium chloride were used for evaluating gave the percentage moisture lost^13^. Mean of five readings were taken. Surface pH was measured by placing pH paper on the buccal films placed on agar plate prepared from 2% w/v of agar in isotonic phosphate buffer pH 6.75^11^. Folding endurance was evaluated by counting the number of times the film could be folded at the same place without breaking or 300 times, which ever occurred earliest^11^. *In vitro* residence time was determined using locally modified USP disintegration apparatus. The medium was made of 800 ml of isotonic phosphate buffer (IPB) pH 6.8 maintained at 37°. Three cm length of rat intestinal mucosa (any part after duodenum but before ceacum) was cut and glued to the surface of a glass slab, vertically attached to the apparatus. The mucoadhesive film was brought into intimate contact with mucosal membrane. The glass slab, attached vertically was allowed to move up and down so that the patch was completely immersed in the buffer solution at the lowest point and out at the highest point. The time necessary for complete erosion or detachment of the patch from mucosal surface was recorded^3^. NTD content in one cm^2^ film was extracted using 10 ml methanol and reduced by treating the extract with concentrated hydrochloric acid (HCl) and zinc dust for 30 min. It was filtered in to a 100 ml volumetric flask and volume was adjusted with 0.1 N HCl. To 1 ml of suitably diluted solution, 1 ml each of 5 N HCl, 0.1% w/v of sodium nitrite solution, 0.5% w/v of ammonium sulfamate solution and 0.1% w/v of N-(naphthyl)ethylenediamine dihydrochloride (NED reagent) were added, mixed and volume was adjusted to 10 ml using 0.1 N HCl. The pink colour developed was at 555 nm against reagent blank in UV spectrometer^14^. Mean value of three films was taken as the amount of nitrendipine present in 1 cm^2^. The results of all the above mentioned tests are shown in Table 2. *In vitro* drug release was executed by placing one cm diameter film on egg membrane acting as semipermeable membrane. This was tied to an open ended cylinder and placed in such a way that the film had dipped into receptor fluid containing 100 ml of IPB pH 6.6. These were assembled on magnetic stirrer. The release study was performed at 37±1° for 2 h^3^. Samples were withdrawn every 15 min and analyzed spectroscopically at 235 nm. Mean of triplicate readings were taken. Graphical representation of the release of tested formulations has been shown in fig. 1. *Ex vivo* permeation through excised porcine buccal mucosa (procured from local slaughter house) was studied using Franz diffusion apparatus. A one cm^2^ film of each formulation under study was placed on the buccal mucosa and the side exposed was covered with aluminum foil as backing membrane. Receptor compartment contained 15 ml of IPB pH 6.6. The cell contents were stirred using magnetic bead at 37±1°. Samples were withdrawn at regular intervals for 2 h and analyzed using UV spectrophotometer at 235 nm.

**Fig. 1:**
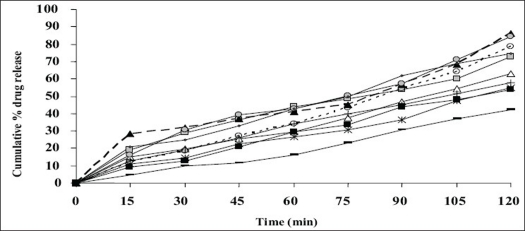
Comparative *In vitro* drug release profile *In vitro* drug release was determined in isotonic phosphate buffer (IPB)at pH 6.6 from formulations F-1 (-△-), F-2 (-□-). F-3 (--▲--), F-4 (…○…), F-5 (-*-), F-6 (-○-), F-7 (-+-), F-8 (-▪-), F-9 (-■-) and F-10 (…▬…)

**TABLE 2:** PHYSICOCHEMICAL EVALUATION OF NITRENDIPINE BUCCAL FILMS

Formulation	Weight (mg)	Thickness (μm)	% moisture		Folding endurance time(min)	*In vitro* residence	NTD content (mg/cm^2^)

			Absorbed	Lost			
F-1	117.0±1.4	208±01	8.48±0.16	20.07±0.07	357±5	172.7±1.7	9.65±0.11
F-2	167.0±2.2	313±14	4.31±0.11	16.13±0.04	366±5	187.0±3.3	10.01±0.34
F-3	236.7±2.5	326±47	5.72±0.16	9.96±0.06	309±3	90.7±1.7	9.32±0.18
F-4	138.3±1.3	185±17	10.35±0.06	3.82±0.17	309±5	195.0±5.1	8.91±0.05
F-5	142.0±1.4	175±07	3.17±0.24	4.57±0.04	274±3	191.7±4.5	9.13±0.11
F-6	346.3±3.4	498±17	2.35±0.05	6.25±0.09	198±3	85.0±1.6	9.00±0.12
F-7	157.7±2.1	270±47	7.17±0.07	10.08±0.13	290±5	158.0±3.4	9.14±0.12
F-8	118.7±2.0	251±33	7.31±0.14	19.30±0.03	481±3	90.7±1.7	9.66±0.03
F-9	115.0±1.6	191±07	5.57±0.05	20.09±0.10	392±4	180.0±4.0	9.85±0.05
F-10	128.0±2.2	224±17	7.69±0.36	21.30±0.03	401±7	239.3±2.5	8.79±0.02

NTD is nitrendipine

Simple matrix type of buccal films was prepared by solvent casting technique. The physicochemical characteristics and bioadhesive performance of all the formulations given Table 2, shows that all the formulations were smooth, flexible, yellow in colour (may be due to the dispersed drug) and elegant in appearance. They were all non-sticky except for F-3 containing 10% w/v of HPC. The high concentration, of 10%w/v of hydrophilic HPC could be the reason for its stickiness. Weight of the films ranged from 115.0±1.6 mg to 346.3±3.4 mg and thickness was found between 175±7 μm to 498±17 μm. The variations in weight and thickness among the formulations may be the effect of difference in molecular weight and proportion of the polymer used in the films. The percentage moisture absorption was observed to range between 2.35±0.05 and 10.35±0.06%, while the moisture loss was 3.82±0.17 to 21.30±0.03%. Percentage moisture absorption is related to the capacity of excipients to absorb water in vapour form. All the excipients used are hydrophilic. It is hypothesized that initial moisture content acts as deciding factor in moisture absorption. Hence the high moisture absorbing capacity detected in F-4. The other films have initially high moisture content as is evidenced by percentage moisture loss. There is inverse relation between these two parameters, higher the percentage moisture loss, lower is the moisture absorption and vice versa. All formulations were of neutral pH formulation and it may be concluded that the films are safe and non-irritating to oral mucosa. The flexibility of the films which is required for their easy handling is given by their folding endurance and it ranged from 481±3 to 198±3. Except F-5 (HPC+CP-934P, 5+1% w/v) and F-6 (sodium alginate 5% w/v), all the films resisted breakage upon folding them for more than 300 times at same place. F-8 made of PVA 3% w/v showed greater endurance. The NTD content was in the range of 8.79±0.13 mg to 10.01±0.34 mg/cm^2^. Though there is less change in the loss of drug among the formulations; more uniformity was seen in F-10. The loss of drug could be attributed to its aqueous insolubility. NTD started settling down from medicated solutions when left undisturbed for removal of air bubbles. Hence the solutions were casted as films contained lesser amount of drug. The viscosity of the solution may affect the settling of NTD. *In vitro* residence time measures the duration of the film adhering to the mucosa and it ranged between 85.0±1.6 min to 239.3±2.5 min. As the polymer particle swell, the matrix experiences an intramatrix force which promoted disintegration of the matrix and leaching of the drug, leaving behind a highly porous matrix. Further water influx weakens the network integrity of the polymer. The structural resistance of the swollen matrices is greatly affected and the erosion of loosely bound gel layer is more pronounced. Erosion of F-6 was the quickest while F-10 was the slowest. PVA based formulation, F-8 dislodged earlier compared to F-9 and F-10 which contained PVP K-30 in addition to PVA. HPC and sodium alginate in combination with SCMC showed better residence time than alone. The integrity of HPC and sodium alginate films was lost early following rapid uptake and swelling compared to other polymers used in the study. This increased the surface area of the polymer, permitting more water influx, results in faster dissolution and erosion from mucosal surface. PVP K-30 in F-9 and F-10 enabled the films to retain on the mucosa for a longer time compared to F-8 which is devoid of it. PVP is a hydrophilic polymer and may have more affinity towards mucin which comprises of 95% water. This may be the reason for longer residence time. *In vitro* release of the drug at the end of 2 h of study was 42.08% w/w for F-10 (PVA+PVP K-30, 2+2% w/v) to 86.40% w/w for F-3 (HPC, 3% w/v). F-6 (sodium alginate, 5% w/v) also showed a comparable release of 84.56% w/w. The higher drug release from F-3 and F-6 may be due to faster swelling and disintegration of polymer network resulting in more void space and more drug release. The residence time and the drug release seem to be directly proportional to each other. The cumulative percentage drug release at the end of 2 h of *in vitro* release study is shown in decreasing order: F-3>F-6>F-4>F-8>F-7>F-2>F-1>F-5>F-9>F-10 (86.40>84.56>8.67>74.33>72.94>63.06>57.69>55.15>54.00>42.08%/w). The plots of percentage drug release against time shown in fig. 1. F-3 showed a good release profile compared to other formulations. From *ex vivo* permeation study (fig. 2), it was observed that maximum percentage of drug diffused through porcine buccal mucosa after 2 h was 60.8% w/w from F-3 (HPC 10% w/v) while minimum was from F-10 (PVA+PVP K-30, 2+2% w/v). The decreasing order of drug release is; F-3>F-6>F-4>F-8>F-7>F-2>F-1>F-5>F-9>F-10 (60.82>58.82>50.29>45.96>41.15>41.1>36.02>33.70>33.31>19.52% w/w). The decrease in drug diffusion observed from *ex vivo* study compared to *in vitro*, may be due to the lesser permeability of porcine mucosa over egg membrane and also the presence of a backing membrane in the *ex vivo* study, which make the release unidirectional. The backing membrane restricting the contact of the film with the receptor fluid to one side alone slows down the water uptake, swelling and disruption of the matrix in turn releasing lesser amount of drug in specified time, compared to the one without the backing membrane. The correlation coefficient values between amounts of drug release versus time were 0.9707, 0.9948, 0.9956, 0.9932, 0.9741, 0.9972, 0.9777, 0.9931, 0.9913, 0.9815, respectively for F-1 to F-10. Significant correlation (99% probability level) was found between drug release versus time. It may be concluded that the release kinetics followed zero order. Though the percentage drug release was higher from F-3, it showed a very short residence time. F-6 also exhibited comparatively better release, but its folding endurance was not appreciable. A moderate release, optimum folding endurance, better residence time and elegant appearance was observed in F-4 made of HPC + CP-934P, 5+2% w/v as film former and glycerin, propylene glycol each 2.5% v/v as plasticizers. Thus it was concluded that F-4 was better formulation for the buccal delivery of NTD.

**Fig. 2:**
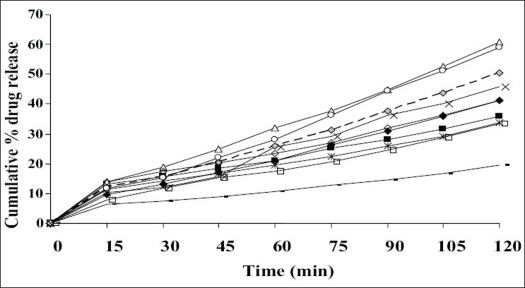
Comparative *ex vivo* diffusion profile Ex vivo drug release was determined in isotonic phosphate buffer (IPB) at pH 6.6 from formulations F-1 (-■-), F-2 (-♦-). F-3 (-△-), F-4 (- -◊- -), F-5 (-*-), F-6 (-○-), F-7 (-◇-), F-8 (-×-), F-9 (-□-) and F-10 (-▬-)
